# Imaging the PM/AICD patient; just because we can, should we?

**DOI:** 10.1186/1532-429X-16-S1-P101

**Published:** 2014-01-16

**Authors:** Robert W Biederman, Mark Doyle, Ronald B Williams, Geetha Rayarao, Diane V Thompson, Moneal Shah, June A Yamrozik

**Affiliations:** 1 Cardiac MRI, Allegheny General Hospital, Pittsburgh, Pennsylvania, USA

## Background

Pacemaker/AICD imaging is infrequently performed on patients in an MRI environment. When the risk justifies the end, however, consideration to perform 'high-risk' scanning can be made on a case-by-case basis but typically with trepidation. However, an important issue has surfaced, "Is the MRI scan adding valuable and irrefutable information to warrant such risk?

## Methods

Over 3 years, 48 patients were imaged via MRI/CMR (1.5T GE, Milwaukee, WI). Specifically: 8 AICD, 7 AICD/PM, 2 retained lead, 2 REVO and 29 dual chamber PM. Specific criteria were followed for all pts to objectively define whether final diagnosis provided *additional *information towards pt care. A checklist of 3 questions was answered following scan interpretation by both the technologist and performing MRI physician: 1) Did the diagnosis change? 2) Did the MRI provide additional information to the existing diagnosis? 3) Did patient management change? If 'Yes' was answered to any of the above questions, it was considered that the MRI scan was of value to patient diagnosis and/or therapy.

## Results

All patients completed the procedure with no death, VT/VF, power-on-reset or adverse events. The PM/AICD was interrogated after the scan by EP/cardiologist to determine changes in impedance, amplitude or threshold. No clinically meaningful changes occured and no post-procedure revisions to generator/lead or parameters were required. Avg MRI: 20 ± 55 min. Regarding the population, 36/48 (75%) were neurology/neurosurgery and 12(25%) were cardiac/vascular cases. After reviewing the 36 neurology cases and comparing the results from prior studies (CT, angio, EEG and/or myelogram), 16/35 (45%) MRIs not only provided additional information but changed the original diagnosis and, in turn, the course of medical treatment. In 8 pts (22%), the MRI provided additional information to the original diagnosis. Thus, a total of 24 pts (67%) demonstrated MRI scanning was of value to the final diagnosis. In 12/36(33%) pts, MRI did not provide further information but confirmed original diagnosis. The 12 cardiac cases were also compared to prior studies (catheterization, TEE, TTE and/or stress) and in 4 pts (33%), the MRI provided additional information to change the original diagnosis and also pt management. The remaining 8 (67%) revealed extra information was gathered via CMR. In essence, 100% of the cardiac population benefited by having a CMR exam performed.

## Conclusions

The use of PM/AICD imaging in MRI remains controversial but as the lead/generator technology has improved and increased confidence in its use is found. We show that MRI/CMR procedures on carefully selected patients with PM/AICDs are beneficial and substantially add valuable, often irrefutable information to pt diagnosis and subsequent management We propose that not only are PM/AICDs safe when imaged properly but potentially no longer 'forbidden' in the MRI environment. Moreover, there can be frequent and marked life-altering and life-saving consequences.

## Funding

N/A.

